# Comparative study of the osteogenic potential of mesenchymal stem cells derived from different sources

**DOI:** 10.4317/jced.53957

**Published:** 2018-01-01

**Authors:** Iman M. Aboushady, Zeinab A. Salem, Dina Sabry, Abbas Mohamed

**Affiliations:** 1 MD, MS, Lecturer of oral biology, Department of Oral Biology, Faculty of Oral and Dental Medicine, Cairo University; 2 MD, MS, Professor of Medical Biochemistry and Molecular Biology, Department of Medical biochemistry and molecular biology, Faculty of medicine, Cairo University; 3 MD, MS, Lecturer of Medical Biochemistry and Molecular Biology, Department of Medical biochemistry and molecular biology, Faculty of medicine, Cairo University

## Abstract

**Background:**

Mesenchymal stem cells (MSCs) can regenerate missing tissues and treat diseases. Hence, the current work aimed to compare the proliferation rate and the osteogenic differentiation potential of bone marrow MSCs (BMSCs), gingival MSCs (GMSCs) and submandibular MSCs (SMSCs).

**Material and Methods:**

MSCs derived from bone marrow, gingiva and submandibular salivary gland were isolated and cultured from rats. The proliferation capacity was judged by MTT proliferation Assay. Osteogenic differentiation was assessed by Alzarin red stain and quantitative RT-PCR was performed for Runx-2 and MMP-13.

**Results:**

The highest significant proliferation was estimated in the BMSCs compared to GMSCs and SMSCs (*p*-value was < 0.01). All studied cell types formed mineralized nodules as stained with Alizarin Red stain at the 3rd passage of differentiation. However, BMSCs seemed to generate the highest level of mineralization compared to GMSCs and SMSCs. RT-PCR revealed that the expression of Runx-2 and MMP-13 mRNAs was significantly increased in the BMSCs compared to GMSCs and SMSCs (*p*-value was < 0.01).

**Conclusions:**

BMSCs displayed maximum osteogenesis results followed by the GMSCs and lastly by the SGSCs. Thus, it could be recommended that GMSCs can be used as a second choice after BMSCs when bone tissue reconstruction is needed.

** Key words:**Mesenchymal stem cells, osteogenic differentiation, Runx-2, MMP-13.

## Introduction

Mesenchymal stem cells (MSCs) are the most promising stem cells for clinical applications; they were originally found in the bone marrow, and have also been isolated from many other tissues as skin, adipose tissue and various dental tissues (1,2). Human bone marrow MSCs from the bone marrow (BMSCs) were distinguished as multipotent stem cells by signifying their separation, growth in culture and directed differentiation to osteogenic, adipogenic and chondrogenic lineages (3). Stem cell plasticity was clarified by the capability of MSCs to differentiate into lineages that are not typical mesenchymal derivatives (4).

Regarding dental originated sources, gingival MSCs (GMSCs) comprise more interesting alternatives to the other dental MSCs as they are much easier to get from the clinically resected gingival tissues. Therefore, it is of great concern to prove the multiple differentiation potentials of GMSCs for possible tissue engineering applications (5). GMSCs have an obvious osteogenic potential. GMSCs from all biopsies were successfully isolated, characterized and expanded to clinically significant numbers (>1 9 107 cells) in 3–5 passages. Multilineage, including osteogenic, differentiation in standard culture was confirmed by staining assays and gene expression (6). The salivary glands derived from the endoderm and consist of acinar and ductal epithelial cells. Salivary gland stem/progenitor cells (SMSCs) were isolated from the rat submandibular glands and investigated that the cells are rapidly proliferative and express acinar, ductal and myoepithelial cell lineage markers (7). We investigated and compared the proliferation rate and the osteogenic differentiation potential of different adult mesenchymal stems from rat bone marrow, gingival and salivary glands.

## Material and Methods

-Ethical Statement

All experiments will be conducted in the animal house of the Faculty of Medicine, Cairo University, Egypt according to the recommendations and approval of the Ethics Committee on animal’s experimentation of the Faculty of Oral and Dental Medicine, Cairo University.

-Experimental procedures

A total of six rats were used in the current study in order to obtain the bone marrow, gingival samples and the submandibular salivary gland samples.

All animals will be euthanized by intracardiac overdose of sodium thiopental.

-Bone marrow mesenchymal stem cells

Bone marrow cells were flushed from tibia and fibula with phosphate-buffered saline (PBS) containing 2 mM EDTA. Over 15 ml Ficoll-Paque (Gibco-Invitrogen), 35 ml of the diluted sample was carefully layered, centrifuged for 35 minutes at 400xg rpm and the upper layer was aspirated leaving undisturbed mononuclear cell (MNC) layer at the interphase. This MNC layer was aspirated, washed twice in PBS containing 2 mM EDTA and centrifuged for 10 minutes at 200xg rpm at 10 ºC. The cell pellet was re-suspended in a final volume of 300 μl of buffer. Isolated MSCs were cultured on 25 ml culture flasks in minimal essential medium (MEM) supplemented with 15% fetal bovine serum (FBS) and incubated at 37°C and 5% CO2. Adherent MSCs were maintained in culture media MEM supplemented with 15% FBS, 0.5% penicillin, and streptomycin at 37°C in 5% CO2 incubator for 10-14 days until reaching 70-80% confluence (8). Cultures confluence was monitored by inverted microscope software image analysis and a digital camera was used for capturing images.

-Gingival mesenchymal stem cells

Gingival tissues were dissected from. Gingival stem cells were isolated by enzymatic digestion with Collagenase II (Sigma-Aldrich) and incubated for 2 hours at 37°C (9). Cells were plated in 25 ml culture flasks and proliferated in minimal essential medium (MEM) (Gibco-Life Technologies) supplemented with 15% fetal bovine serum (FBS), 1× Pen/Strep antibiotics (Invitrogen) and incubated at 37°C and 5% CO2. Cell Culture media were changed twice a week; with supplementation of L-Ascorbic Acid 2-Phosphate (50 μg/mL; Sigma-Aldrich) and they were monitored for 70 to 80% confluence by using inverted microscope software.

-Salivary mesenchymal stem cells

Primary salispheres were cultured as published previously (10). In brief, cell suspensions were prepared first by mechanical disruption with sterile scissor followed by enzymatic digestion with collagenase II (0.63 mg/ml; Gibco). After washing thoroughly, cell suspensions were resuspended in DMEM/F12 medium containing 1× Pen/Strep antibiotics (Invitrogen), EGF (20 ng/ml; Sigma-Aldrich), FGF2 (20 ng/ml; Sigma-Aldrich), insulin (10 μg/ml; Sigma-Aldrich), and dexamethasone (1 μM; Sigma-Aldrich), at a density of 400,000 cells per well of a 12-well plate. Cell culture media was changed twice a week, until they reached 70 to 80% of confluence. Inverted microscope software image analysis was used to assess vitality and confluence of cultured cells.

-MTT proliferation Assay

The MTT Reagent and Detergent Solution were obtained from TACSTM TREVIGEN® supplied ready for use. Cells (103-105) were cultured in 100 µl of culture medium in a flat-bottomed 96 well plate (tissue culture grade). The MTT reagent and detergent reagent were added to each well. The absorbance in each well was measured at a range from 490 to 630 nm using an enzyme-linked immunosorbent assay plate reader (Dynatech MRX 5000; Dynex).

-Osteogenic differentiation

At three passage of subcultures, 2×103 of BMSCs, 2×103 GMSCs and 2×103 of SMSCs were plated in each well of a flat-bottom 24-well plate with 1 mL of culture medium. Osteogenic differentiation and mineralization was initiated 24 h after plating by replacing culture medium with DMEM supplemented with 10% FBS, 100 nM dexamethasone, 50 μg/mL ascorbic acid-2-phosphate. For mineralization, 10 mM β-glycerophosphate was added to osteogenic medium. Staining with Alzarin red was applied to each culture to assess BMSCs, GMSCs and SMSCs differentiation into osteoblasts (11).

-Quantitative real time PCR

All studied cells were collected and subjected to RNA extraction using Qiagene cells/tissue extraction kit (Qiagene) according to manufacture instructions. The mRNA expression level was quantified by qRT–PCR. 1000 ng of the total RNA from each sample were used for cDNA synthesis by reverse transcription using High capacity cDNA Reverse Transcriptase kit (Applied Biosystem). The cDNA was subsequently amplified with the Syber Green I PCR Master Kit (Fermentas) in a 48-well plate using the Step One instrument (Applied Biosystem) as follows: 10 minutes at 95 ºC for enzyme activation followed by 40 cycles of 15 seconds at 95ºC, 20 seconds at 55 ºC and 30 seconds at 72 ºC for the amplification step. We used 1 μM of both primers specific for each selected gene including Runt-related transcription factor-2, matrix metalloproteinase-13 (RUNX-2, MMP-13) and GAPDH. Primers sequence specific for each gene was demonstrated in  Table 1. Changes in the expression of each target gene were normalized relative to the mean critical threshold (CT) values of GAPDH housekeeping gene by the ΔΔCt method.

**Table 1:**
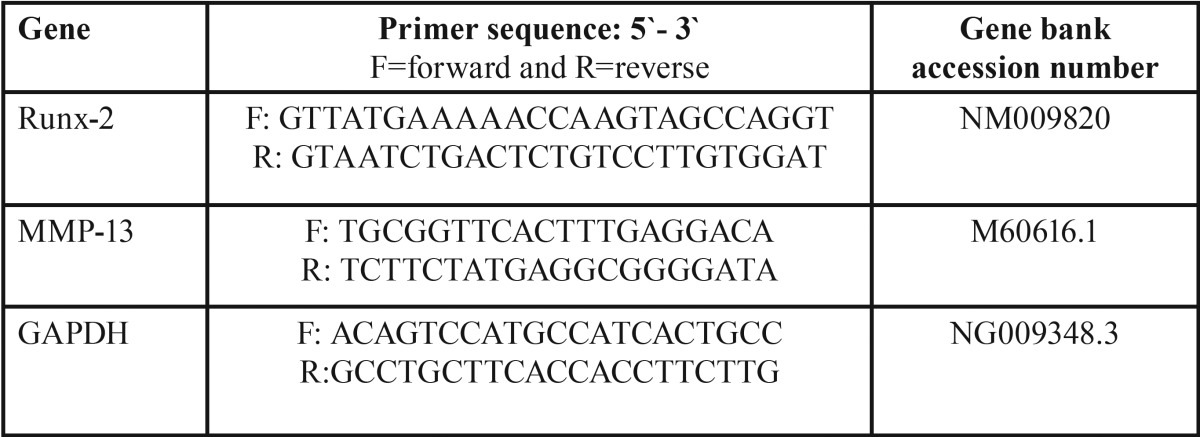
Primers sequence specific for each studied gene.

-Statistical analysis

Data were statistically described in terms of mean ± standard deviation (± SD), median and range, or frequencies (number of cases) and percentages when appropriate. Comparison of numerical variables between the study groups was done using Kruskal Wallis test with post hoc multiple 2-group comparisons. *P* values less than 0.05 was considered statistically significant. All statistical calculations were done using computer program SPSS (Statistical Package for the Social Science; SPSS Inc., release 15 for Microsoft Windows 2006).

## Results

-Isolation and Culture of BMSCs, GMSCs and SMSCs

No differences were observed in the morphological characteristics of BMSCs, GMSCs and SMSCs at one and two weeks of culture. All the three groups achieved the spindle fusiform shaped like cells in morphology.

-MTT proliferation Assay

Measuring the MTT color absorbance among the three studied groups revealed that the highest significant proliferation was observed at two weeks culture in BMSCs (2.416±0.744), followed by GMSCs (1.281±0.577) and finally SMSCs (0.226±0.0225) ( Table 2). At 7&14 days culture there was a statistically highly significant difference between all the studied groups as the *p*-value was < 0.01.

**Table 2:**
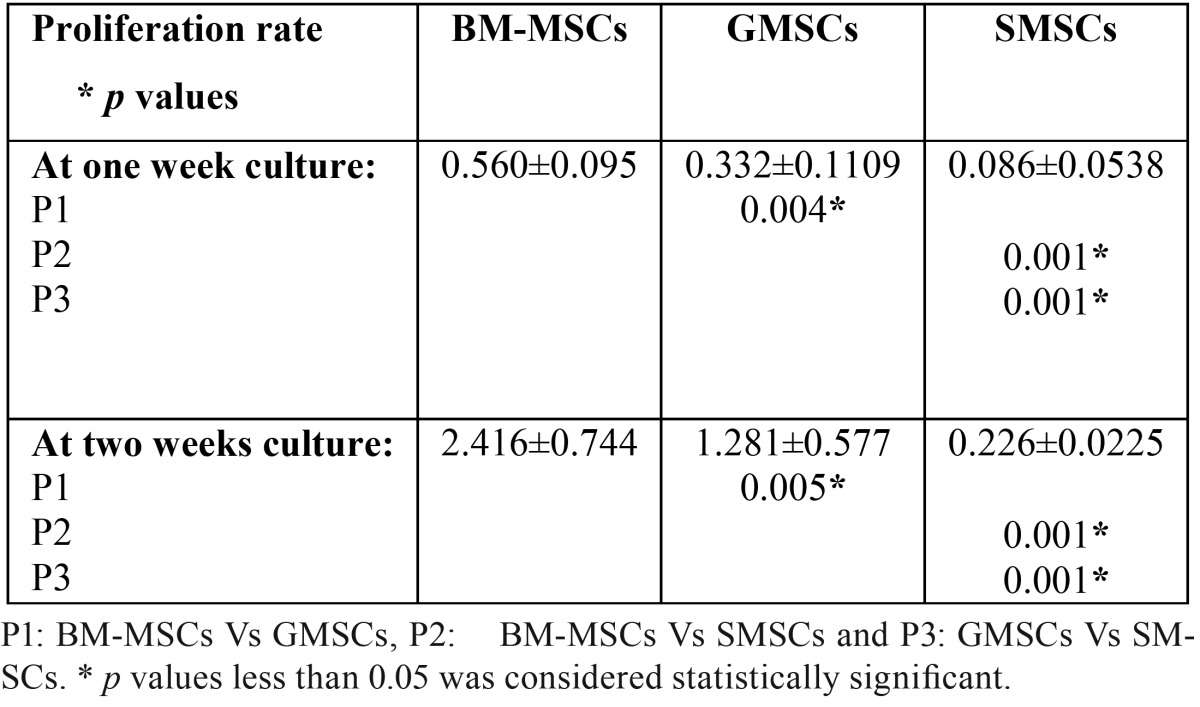
Proliferation rate in the different studied groups (mean±SD).

-Osteogenic differentiation

**Figure 1:**
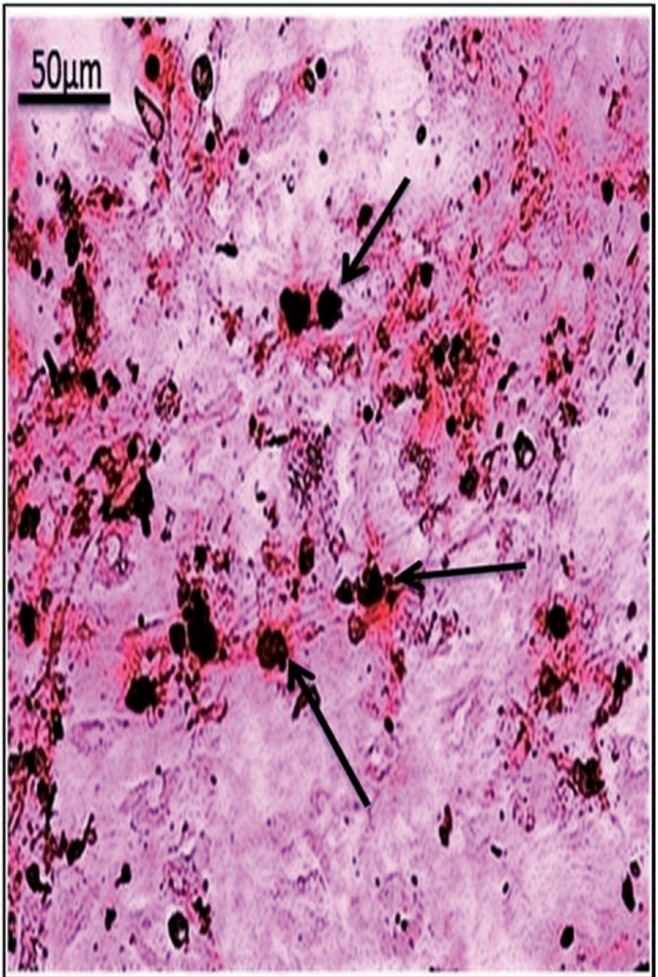
BM-MSCs at 3rd passage differentiated into osteoblasts and stained with Alzarin red. (arrows showed higher calcium mineralization deposits compared to other groups).

**Figure 2:**
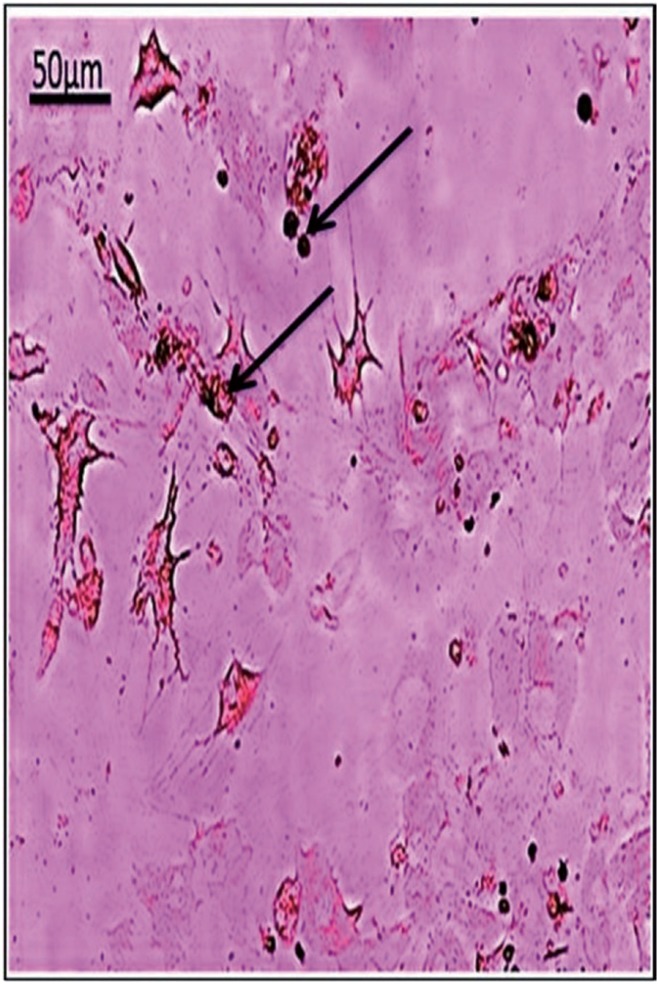
GMSCs at 3rd passage differentiated into osteoblasts and stained with Alzarin red. (arrows indicating moderate calcium mineralization deposits).

**Figure 3:**
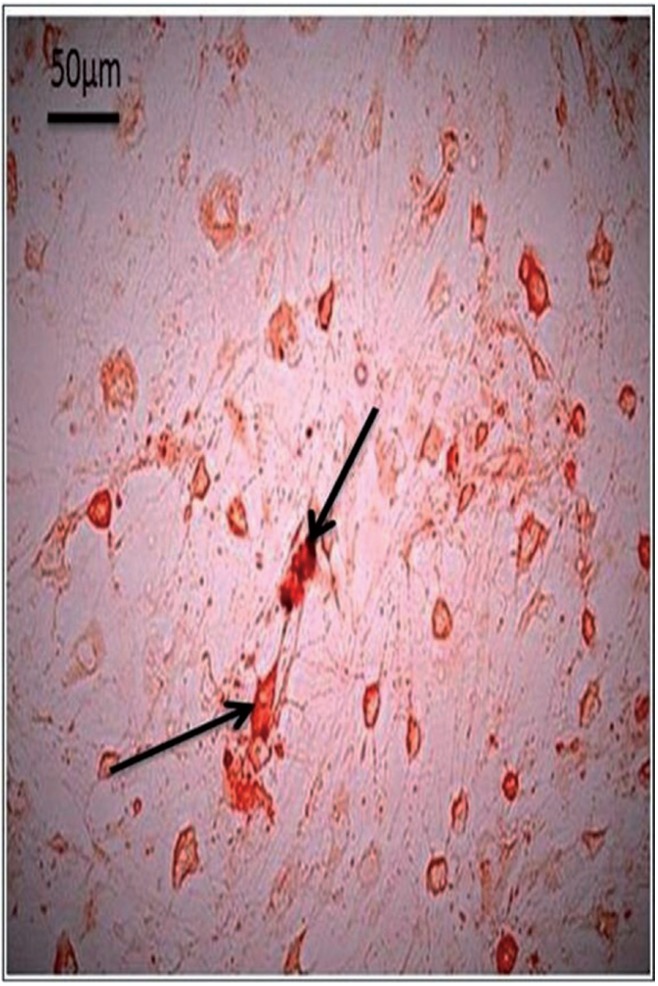
SMSCs at 3rd passage differentiated into osteoblasts and stained with Alzarin red. (arrows indicating lower calcium mineralization deposits).

Osteogenic differentiation and mineralization were evidenced by calcium deposits which formed orange red facets around the differentiated cells. BM-MSCs, GMSCs and SMSCs formed mineralized nodules with Alizarin Red stain at the 3rd passage differentiation (Figs. 1-3 respectively). The highest calcium deposits were observed in BM-MSCs compared to GMSCs and SMSCs.

-Quantitative RT-PCR results

Quantitative RT-PCR revealed that the expression of Runx-2 and MMP-13 mRNAs was increased in the BM-MSCs followed by the GMSCs group and lastly the SMSCs ( Table 2, Table 3).

**Table 3:**
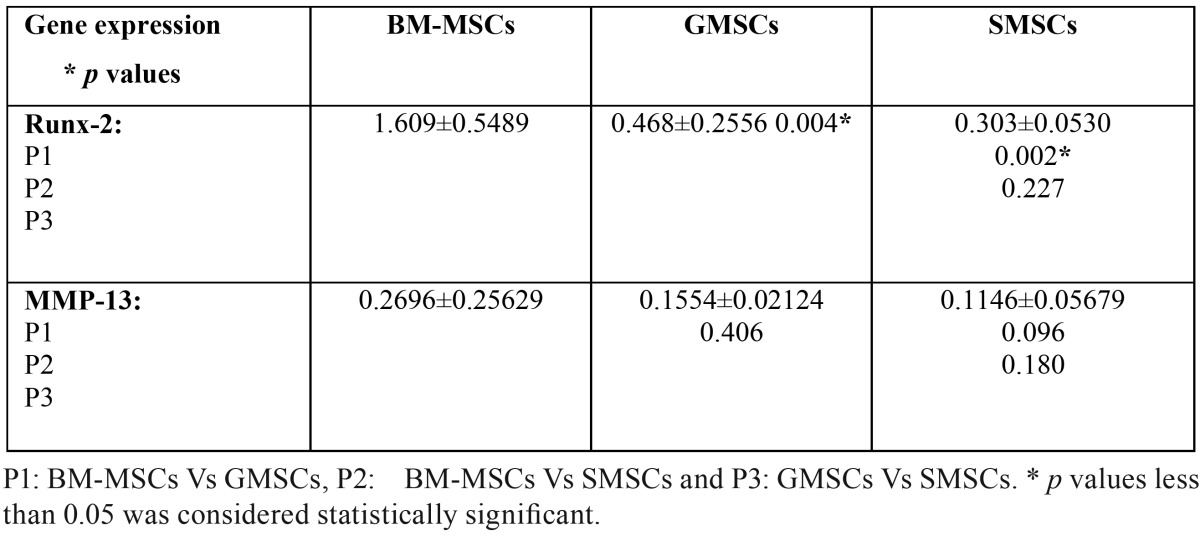
Quantitative RT-PCR results of RUNX-2 and MMP-13 genes expression (mean±SD).

Comparing the mean values ± SD for the three studied groups regarding the proliferation results at 7&14 days in culture and PCR results of Runx-2 showed that there was a statistically highly significant difference between the studied groups as the *p*-value was < 0.01; while the PCR results of MMP-13 were statistically not significant as the *p*-value was > 0.05.

On the other hand, 2-groups comparison of the mean values ± SD as regard the proliferation results at 7&14 days culture showed a statistically highly significant difference between each pair of the studied groups as the *p*-value was < 0.01.

Concerning the PCR results of Runx-2; a statistically highly significant difference occurred between the BM-MSCs & GMSCs groups and BM-MSCs & SMSCs groups; while the difference between the GMSCs & SMSCs groups was statistically not significant as the *p*-value was > 0.05. Besides, comparing the PCR results of MMP-13 between each pair of the studied groups revealed a statistically non-significant difference as the *p*-value was > 0.05 ( Table 2, Table 3).

## Discussion

Stem cells are expected to provide a novel alternative to regenerate large defects in periodontal tissues and alveolar bone (12). In the maxillofacial region, adult mesenchymal stem cells (MSCs) have been characterized in several oral and para-oral tissues, which suggests that these tissues could represent rich sources of stem cells, and in turn, could be used as alternatives for other conventional stem cell sources like bone marrow for example. This, in turn, has motivated us to carry out an in vitro study comparing the proliferation rate and osteogenic potential of MSCs obtained from various sources: bone marrow, gingiva and submandibular salivary glands. BMSCs can be easily isolated from the bone marrow of iliac crest by physicians, but the bone marrow aspiration procedure is invasive for the donors. However, the stem cells most commonly used to date for bone regeneration in dental patients are BMSCs obtained from the iliac crest, owing to their great potential for bone regeneration (13). Other stem cells in the oral mucosa have been identified in the lamina propria of the gingiva, which attaches directly to the periosteum of the underlying bone with no intervening submucosa (14). The gingiva overlying the alveolar ridges and the retromolar region is frequently resected during ordinary dental treatments and thus, can often be obtained as a discarded biological sample. GMSCs exhibited clonogenicity, self-renewal and a multipotent differentiation capacity similar to that of BMSCs. However, they proliferate faster than BMSCs, display a stable morphology and don’t lose their MSC characteristics with extended passaging (15). The multipotency of GMSCs and their ease of isolation, clinical abundance and rapid in vitro expansion provide a great advantage for this source as a stem cell source for potential clinical applications.

Concerning stem cells in adult salivary gland, they are expected to be useful for autologous transplantation therapy in case of tissue engineered-salivary glands or direct cell therapy. SMSC could be directed to differentiate into adipogenic, osteogenic and chondrogenic cells (16). Long-term intake of glucocorticoids would cause osteoporosis and decreased osteoblastic activity which ultimately can result in Cushing’s syndrome (17). In addition, dexamethasone when used for a long time would be toxic to the differentiated osteoblasts, hence, the cells have to be soon harvested and used after full differentiation (18). Osteogenic differentiation media contain dexamethasone, ascorbic acid and β- glycerophosphate (β-GP) was used to support the osteogenic differentiation of mesenchymal stem cells in vitro. However, the exact mechanism by which β-glycerophosphate can induce mineralization remains unclear, but it is believed that alkaline phosphatase can hydrolyze organic phosphate and release inorganic phosphate which in turn can promote mineral deposition on the surface of tissue culture plastic and other materials (19). Organic phosphates are known to aid osteogenesis by starting mineralization in cell cultures and they were suggested to modulate osteoblastic activities by promoting a bone-like mineral phase (20). Mesenchymal stem cells are shown to undergo osteogenic differentiation when they are grown on mineralized surfaces which might be owing to the presence of osteopontin which strongly adsorbs to the charged mineral phases created by presence of β-glycerolphosphate and other organic phosphates (21). Mineralization is not solely affected by the exogenously-added free phosphate groups, but also cell density plays an important role in mineralization. Higher MSCs seeding densities would lead to significantly more mineralization, demonstrating that a certain threshold cell density should be reached before mineralization. Furthermore, cultures that were allowed to concentrate their soluble products in the media produce more mineralized matrix indicating an autocrine or paracrine role of factors synthesized by MSCs which are undergoing osteogenic differentiation (22). Consequently, these results could explain our findings, where the greatest amount of mineralization; as detected by the alizarin red stained nodules, was observed among the BMSCs group as compared to the other two groups, which could be owing to the higher cell density and thus higher mineralization.

Regarding the in vitro MTT proliferation results for the cultured cells; the significant proliferation was noticed among the BMSCs. Maximum proliferation was achieved at 14 days for the three studied groups. A statistically highly significant difference was noticed among the three studied groups as the *p*-value was < 0.01.

The core binding factor (Cbfa1) gene, also referred to as runt-related transcription factor-2 (Runx-2), and acute myelogenous leukemia factor (AML-3), encodes a transcription factor that shares homology with the Drosophila segmentation gene product Runt (23,24) and plays a key role in osteoblastic differentiation (25). The role of Runx-2 protein in bone formation has been demonstrated in Runx-2 deﬁcient mice which manifest a total absence of osteogenesis (25). In addition, genetic mutations in Runx-2 gene have been associated with multiple skeletal abnormalities in patients with cleidocranial dysplasia syndrome (26). Hence, in the present investigation, the increased expression of Runx-2 among the BMSCs group could suggest more enhanced osteoblastic differentiation in this group. A statistically highly significant difference in the expression of Runx-2 occurred among the three studied groups as the *p*-value was < 0.01.

Matrix Metalloproteinase 13 (MMP13, also called collagenase-3) is a member of the large family of matrix metalloproteinases. This is a family of extracellular matrix-degrading enzymes having several common structural features. They are endopeptidases which regulate cell growth, migration, and extracellular matrix remodeling (27).

MMP13 has been considered to have an essential role in bone biology owing to its exclusive presence in the skeleton during development. MMP13 plays an important role in the degradation of components of the extracellular matrix, particularly collagens. It degrades mainly collagen type II, but also collagens type I, III, and X, which are the major components of cartilage and bone (28). MMP13 knockout mice fail to undergo normal ossification with a delay in ossification at the primary centers (29). Because of these findings, MMP13 is considered important in bone formation and remodeling and hence, it was selected in the present work to investigate the osteoblastic differentiation of the studied MSCs. The current PCR results of MMP-13 revealed that its expression was significantly increased among the BMSCs group compared to GMSCs and to SGSCs. However, the differences among the studied three groups were statistically not significant as the *p*-value was > 0.05.

According to the obtained results in the current investigation, it could be concluded that MSCs from different sources possess an osteogenic potential as they could be differentiated into osteoblasts and could produce mineralized nodules, when cultured in the proper osteogenic medium. This was supported by alizarin red staining results and by RT-PCR examination for the expression of Runx-2 and MMP-13 genes.
